# Role of Chd7 in Zebrafish: A Model for CHARGE Syndrome

**DOI:** 10.1371/journal.pone.0031650

**Published:** 2012-02-20

**Authors:** Shunmoogum A. Patten, Nicole L. Jacobs-McDaniels, Charlotte Zaouter, Pierre Drapeau, R. Craig Albertson, Florina Moldovan

**Affiliations:** 1 Sainte-Justine Hospital Research Center, Montreal, Quebec, Canada; 2 Faculty of Dentistry, University of Montreal, Montreal, Quebec, Canada; 3 Department of Biology, Syracuse University, Syracuse, New York, USA; 4 Department of Pathology and Cell Biology, Faculty of Medicine, University of Montreal, Montreal, Quebec, Canada; 5 Department of Biology, University of Massachusetts, Amherst, Massachusetts, USA; National University of Singapore, Singapore

## Abstract

CHARGE syndrome is caused by mutations in the *CHD7* gene. Several organ systems including the retina, cranial nerves, inner ear and heart are affected in CHARGE syndrome. However, the mechanistic link between mutations in *CHD7* and many of the organ systems dysfunction remains elusive. Here, we show that Chd7 is required for the organization of the neural retina in zebrafish. We observe an abnormal expression or a complete absence of molecular markers for the retinal ganglion cells and photoreceptors, indicating that Chd7 regulates the differentiation of retinal cells and plays an essential role in retinal cell development. In addition, zebrafish with reduced Chd7 display an abnormal organization and clustering of cranial motor neurons. We also note a pronounced reduction in the facial branchiomotor neurons and the vagal motor neurons display aberrant positioning. Further, these fish exhibit a severe loss of the facial nerves. Knock-down of *Chd7* results in a curvature of the long body axis and these fish develop irregular shaped vertebrae and have a reduction in bone mineralization. *Chd7* knockdown also results in a loss of proper segment polarity illustrated by flawed *efnb2a* and *ttna* expression, which is associated with later vascular segmentation defects. These critical roles for Chd7 in retinal and vertebral development were previously unrecognized and our results provide new insights into the role of Chd7 during development and in CHARGE syndrome pathogenesis.

## Introduction

Mutations in the *CHD7* gene (NM_017780) in 8q12.1 were identified as causative for CHARGE (Coloboma, Heart defects, Atresia of the choanae, Retarded growth and development, Genital hypoplasia, Ear anomalies; OMIM # 214800)) syndrome [1], [2], [3], [4], [5]. Heterozygosity for nonsense, deletion or missense *CHD7* mutations is estimated to occur in 60–80% of patients with CHARGE syndrome; these mutations are distributed throughout the coding sequence and do not appear to be correlated with specific aspects of the clinical phenotype [6], [7], [8]. The majority of *CHD7* mutations identified thus far are *de novo*; however, evidence for germline mosaicism has been suggested for families with multiple affected siblings [9] . Children affected by CHARGE syndrome have a variable association of features including ocular deficits, heart malfunctioning, olfactory dysfunction, retarded growth, vestibular dysfunction, cranial nerve anomalies and intellectual disability [2], [4], [10]. All of these characteristics have variable degrees of penetrance, with some being present in virtually all CHARGE patients, whereas others are less frequently observed [11]. Although there is clear evidence that mutations in *CHD7* gene are causative in CHARGE syndrome, the pathogenic mechanisms elicited by these mutations that lead to organs and systems dysfunction are not fully understood.

Chd7 is one of nine members of the chromodomain helicase DNA- binding (Chd) domain family of ATP-dependent chromatin remodeling enzymes [12]. It consists of functional domains such as a chromatin organization modifier, SNF2-related helicase/ATPase and BRK [12], [13]. Recently, DNA-binding sites on chromatin have shown that chd7 binding is correlated to areas of mono- and dimethylated lysine 4 of histone H3 [14], [15]. The *Chd7 Drosophila* ortholog, *Kismet* down-regulates transcriptional elongation by RNA polymerase II through the recruitment of ASH1 and TRX and may be involved in the maintenance of stem cell pluripotency by regulating methylation of histone H3 lysine 27. Chd7 is also implicated in cell fate specification of mesenchymal stem cells [16] . During osteoblast and adipocyte differentiation, Chd7 forms a complex with NLK, SETDB1 and PPAR-γ, then binds to histone H3 at PPAR-γ target promoters and suppresses ligand-induced transactivation of PPAR-γ target genes, which leads to a change in cell fate [16] . Recently, Chd7 has been shown act synergistically with PBAF to promote neural crest gene expression and cell migration [17]. Thus, Chd7 is thought to play a variety of essential roles during development in many species.

The understanding of cause and pathogenesis of many complex human diseases has been improved through the study of model organisms. Zebrafish are a well-established model used to study developmental biology because of their accessibility, optical transparency and rapid development. Over the past decade, the utility of this model organism in investigations of human health and disease has become more explicit. Major advantages of this vertebrate model include a high degree of homology to human genes, as well as conservation of developmental pathways. Zebrafish *chd7* gene is located on chromosome 2 and alignment of the zebrafish genomic sequence with mouse *Chd7* and human *CHD7* shows the gene structure is conserved across species [18]. Given that the zebrafish *chd7* gene is highly similar to the human *CHD7* gene, elucidating roles for Chd7 in zebrafish development could significantly contribute to our understanding of the function of Chd7 in CHARGE syndrome pathogenesis. Therefore, we sought to utilize zebrafish to assess the pathogenic effect of the loss of function of Chd7 and understand the role of the zebrafish homolog of the mammalian *Chd7* during development. We show that *chd7* is robustly expressed in developing zebrafish embryos, particularly in the retina, hindbrain and tail bud. Loss of function of Chd7 results in several defects including abnormal neural development, curvature of the long axis of the body, abnormal cranial neural crest (CNC) development, otolith anomalies, smaller eye and pericardial edema. These defects are similar to the major congenital anomalies associated with human *CHD7* mutations in CHARGE syndrome. Finally, we reveal for the first time a critical role of Chd7 in retinal organization and bone mineralization.

## Results

### *Chd7* transcript is broadly expressed in developing zebrafish

**Figure 1:**
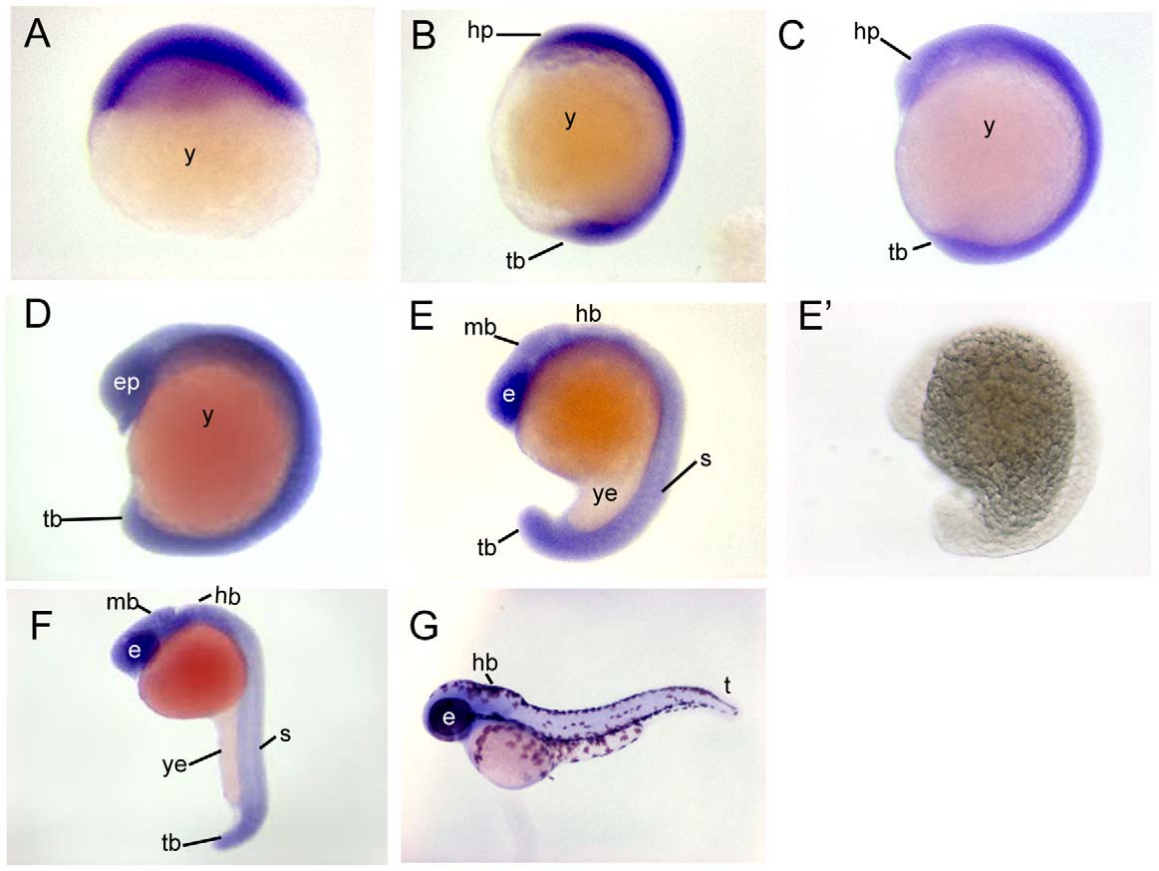
*Chd7* expression patterns during zebrafish embryogenesis. *Chd7* was expressed ubiquitously throughout the embryo during epiboly (**A**), at the 4 somite stage (**B**), and at the 8 somite stage (**C**). (**D**) At the 13 somite stage, expression remained relatively ubiquitous, but with stronger expression noted within the retina and at the perimeters of developed somites. (**E**) At the 18 somite stage, tissue-specific expression was observed in the eyes, brain, somites, and tailbud. This expression pattern remained through 24 hours post fertilization (**F**). (**E′**) No staining occurred when hybridized with a *chd7* sense probe. (**G**) At 48 hours post fertilization, *chd7* expression began to diminish from the body of the zebrafish but remained in the eye and in the brain. e, eye; ep, eye primordium; mb, midbrain; hp, head primordium; s, somite; t, tail; tb, tailbud; y, yolk; ye, yolk extension.

To begin to explore the role of Chd7 in developing zebrafish, we first analyzed the developmental expression of *chd7* mRNA. *In situ* hybridization results showed that *chd7* was expressed relatively ubiquitously throughout early zebrafish embryogenesis (Fig. 1 A–C) until around the 13 somite stage of development when more discrete expression in the eye primordium, and the perimeter of the somites was observed (Fig. 1 D). As somitogenesis continued, *chd7* became highly expressed in the retina as well as in the brain, somites, and tailbud of the embryo (Fig. 1 E–F). Finally at 48 hpf, *chd7* expression in the tail began to diminish, however expression in the brain and in the eye remained strong (Fig. 1 G). The sense probe did not show any *in situ* staining suggesting that there was no non-specific hybridization (Fig. 1 E′).

### Loss of function of Chd7 leads to several and widespread morphological abnormalities

**Figure 2:**
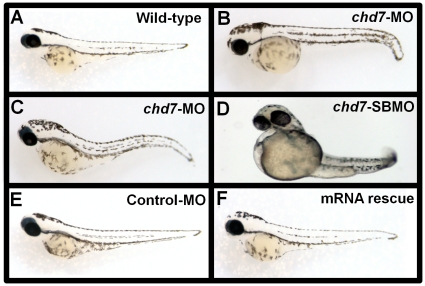
*Chd7*- MO injections and mRNA rescue experiments. Control-MO injected (**E**) *chd7*-MO+*chd7*-mRNA co-injected (**F**) zebrafish showed no phenotypic defects at 48 hpf and were comparable to wild type zebrafish (**A**) at the same age. Embryos injected with 2 ng/nl *chd7*-MO (**B**–**C**), or 2 ng/nl (**D**) *chd7*-SBMO showed several developmental defects.

Next, we depleted zebrafish embryos of Chd7 protein using an antisense morpholino oligonucleotide (MO) designed against the translation initiation sites of chd7 mRNA. Embryos injected with *chd7*-MO exhibited phenotypes marked by curvature of the long axis of the body, a flattening of the head, abnormal tail fins, smaller eyes and heart defects (n = 12, A total of 498 fish used; Fig. 2, 3 A–C). These defects were observed in a dosage-dependent manner (Figure S1). As embryos were injected with increasing concentrations of *chd7*-MO, the resulting defects became more severe (Figure S1 C–D)

**Figure 3:**
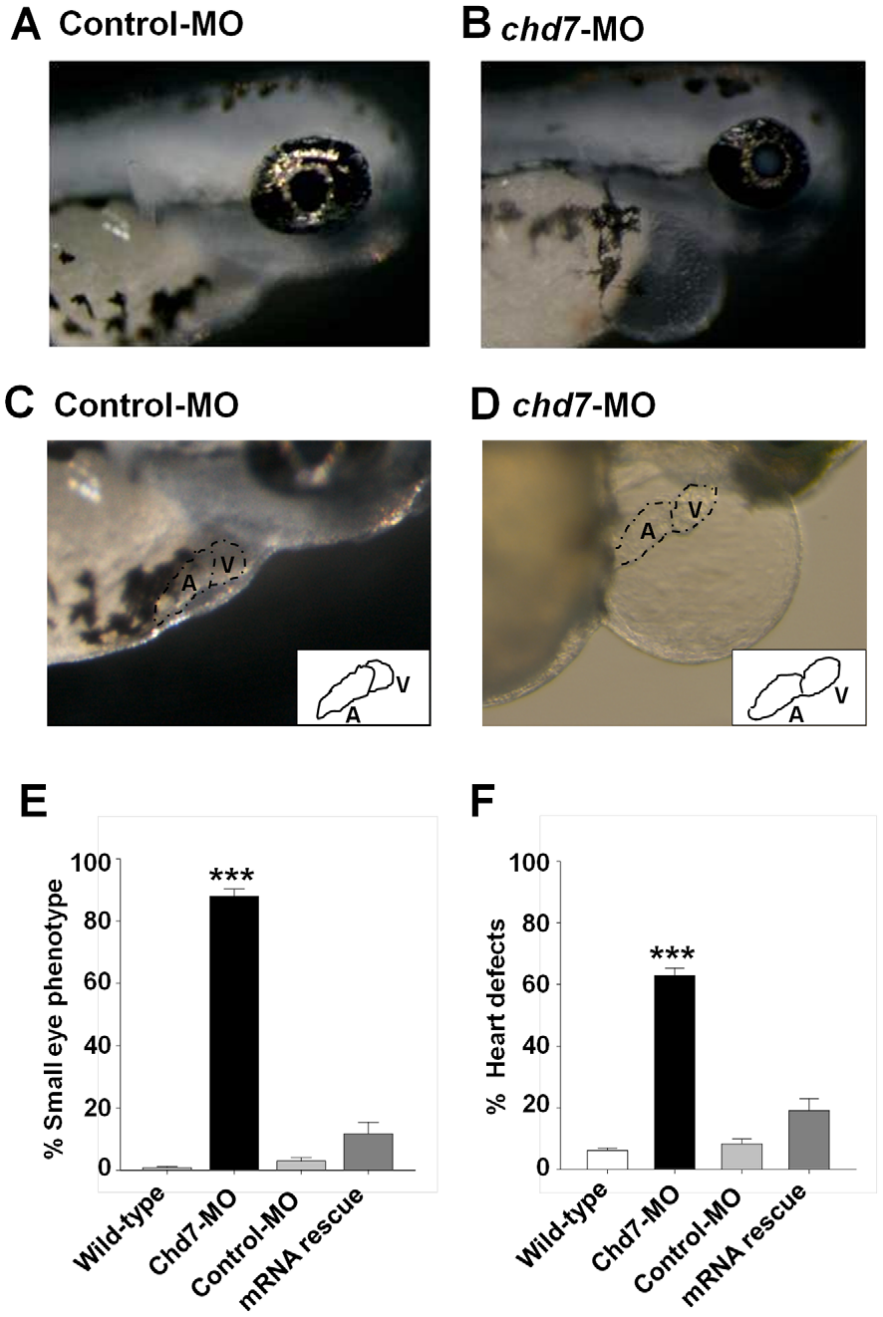
Knockdown of *chd7* affects eye and heart development. (**A–B**) Representative images of eye phenotypes induced by injection of *chd7*-MO in zebrafish embryos (**B**) compared with control embryos (**A**) at 72 hpf. Lateral view with anterior to right. *Chd7* morphants had a reduced ocular size. *Chd7*-MO-injected zebrafish exhibited cardiac anomalies such as dysmorphic heart and tube-like heart shape (**D**) compared with control embryos (**C**). Insets in (**C**) and (**D**) represent an illustration of the heart phenotype in control or *chd7* morphant embryos, respectively. (**E–F**) Bar graphs illustrating the prevalence of eye (**E**) and heart (**F**) phenotypes in wild-type (white bar), *chd7*-MO (black bar), control-MO-injected embryos (grey bar) and mRNA rescue (dark grey bar). ***<0.001, *<0.01. ***<0.001, *<0.01.

To exclude the possibility that this phenotype may have resulted from non-specific or mistargeting effects of the MO, we also designed and injected zebrafish embryos with a *chd7* splice-blocking morpholino (SBMO) and control 5-bp mismatch MO. We recently showed by RT-PCR analysis that the morpholino-targeted region exhibited abnormal splicing of *chd7* in embryos injected with *chd7* SBMO and normal splicing in uninjected and control-MO injected embryos [19]. Embryos injected with the *chd7* SBMO exhibited morphological defects similar to those observed in the *chd7*-MO morphants (Fig, 2 D). On the other hand, all control MO-injected larvae developed normally (n = 12, 436 fish; Fig. 2 E and Fig. 3) and no apparent phenotype was observed when compared to untreated wild-type embryos (n = 12, 510 fish; Fig. 2 A). To further confirm that the lack of Chd7 underlies all the phenotypic alterations observed in *chd7*-MO zebrafish, a rescue experiment was performed. We found that expression of *chd7* mRNA which had no complementary sequence to *chd7*-MO can correct all the developmental defects (n = 6, 249 fish; Fig. 2 E) and no apparent phenotype was observed when compared to control-MO fish (Fig. 2). Thus, the amount of newly-synthesized Chd7 coded by the rescuing mRNA in the initial stages was sufficient to prevent the developmental anomalies imparted by the *chd7*-MO. Altogether these findings were consistent with a reduction in Chd7 expression and they suggest that the phenotype observed with *chd7*-MO-injected embryos was a specific and due to a direct result of Chd7 protein depletion.

We focused the analysis of the loss of function of Chd7 in embryos injected with 2 ng/nl of *chd7*-MO. At 48 hpf, we found that chd7-MO-injected (2 ng/nl) embryos exhibited a small eye phenotype (Fig. 3B,E) compared to control-MO (Fig. 3A,E) and wild-type fish (Fig. 3E). We observed that the hearts of *chd7*-MO-injected embryos were developmentally impaired and have signs of severe pericardial edema compared to controls and wild-type fish (Fig. 3 F). The atrial and ventricular chambers in these fish can be distinguished (Fig 3. D), but these compartments were dysmorphic and more tube-like (inset, Fig. 3 D) than the well-defined and tightly looped chambers of control-MO injected fish and wild-type fish (Fig. 3C). Injection of *chd7* mRNA significantly rescued the morpholino eye and heart phenotypes (Fig. 3E–F).

**Figure 4:**
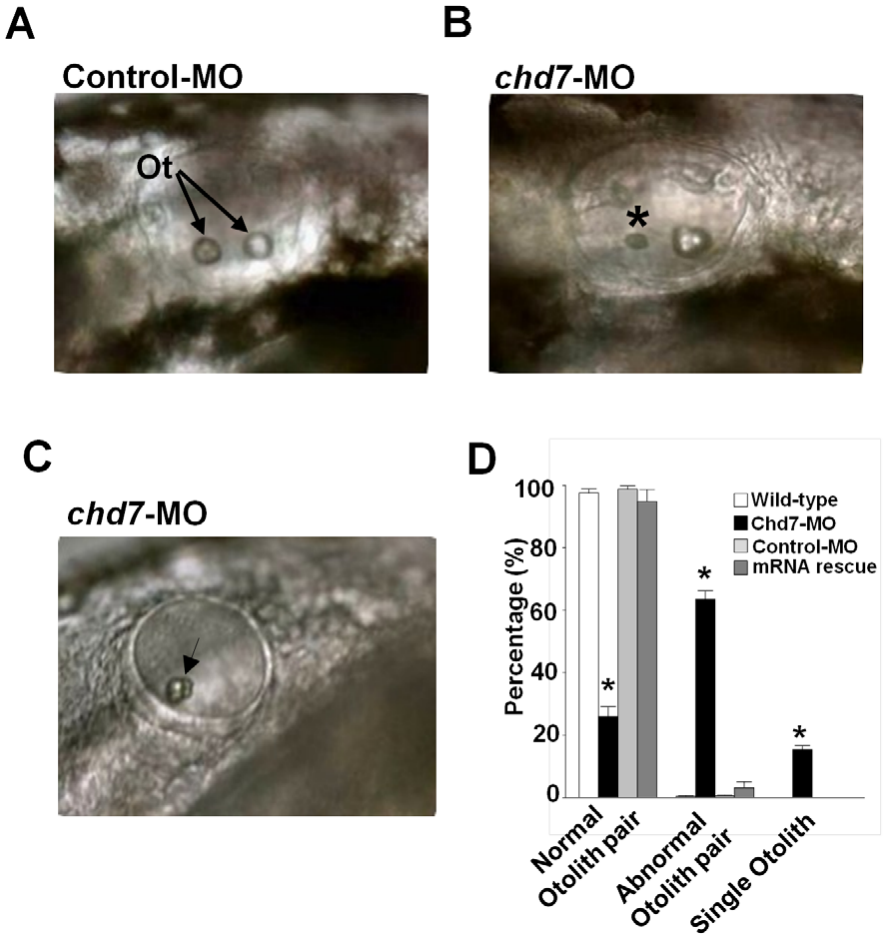
Knockdown of *chd7* affects otolith development. (**A**) The otic vesicle of control-MO-injected fish contains two otoliths, by contrast, larva treated with a morpholino against *chd7* (**B,C**) had smaller otoliths (*) or only a single otolith (Arrow). Lateral views with anterior to the left. Ot, otoliths (**D**) Bar graphs illustrating the prevalence of otolith phenotypes in wild-type (white bar), *chd7*-MO (black bar), control-MO-injected embryos (grey bar) and mRNA rescue (dark grey bar). ***<0.001, *<0.01.

At 48 hpf, *chd7*-MO-injected (2 ng/nl) embryos also displayed a very specific defect in otolith formation (n = 6, 214 fish; Fig. 4B–D). We observed that 63% of the morphants exhibited otoliths that were asymmetric in size (135/214; p = 0.006, ANOVA; Fig. 4 B,D), and 15% possessed only one otolith (32/214; p = 0.008, ANOVA; Fig. 4 C–D). The enlarged or single remaining otolith was often shaped irregularly as opposed to the round and smooth otoliths in control-MO (n = 6, 249 fish; Fig. 4 C), wild-type embryos or mRNA rescue embryos (Fig. 4-D). In addition, the general size and morphology of the ear and the formation of semicircular canals were affected in morphants with a single otolith. Fish with defective otoliths also had a pronounced curvature of the body axis, usually remained on their sides at the bottom of their dish, and exhibited a circling swimming behavior upon a touch (tail tap) or acoustic (dish tap) stimulation (data not shown).

### Chd7 plays critical roles in primary axis development and vertebral mineralization

**Figure 5:**
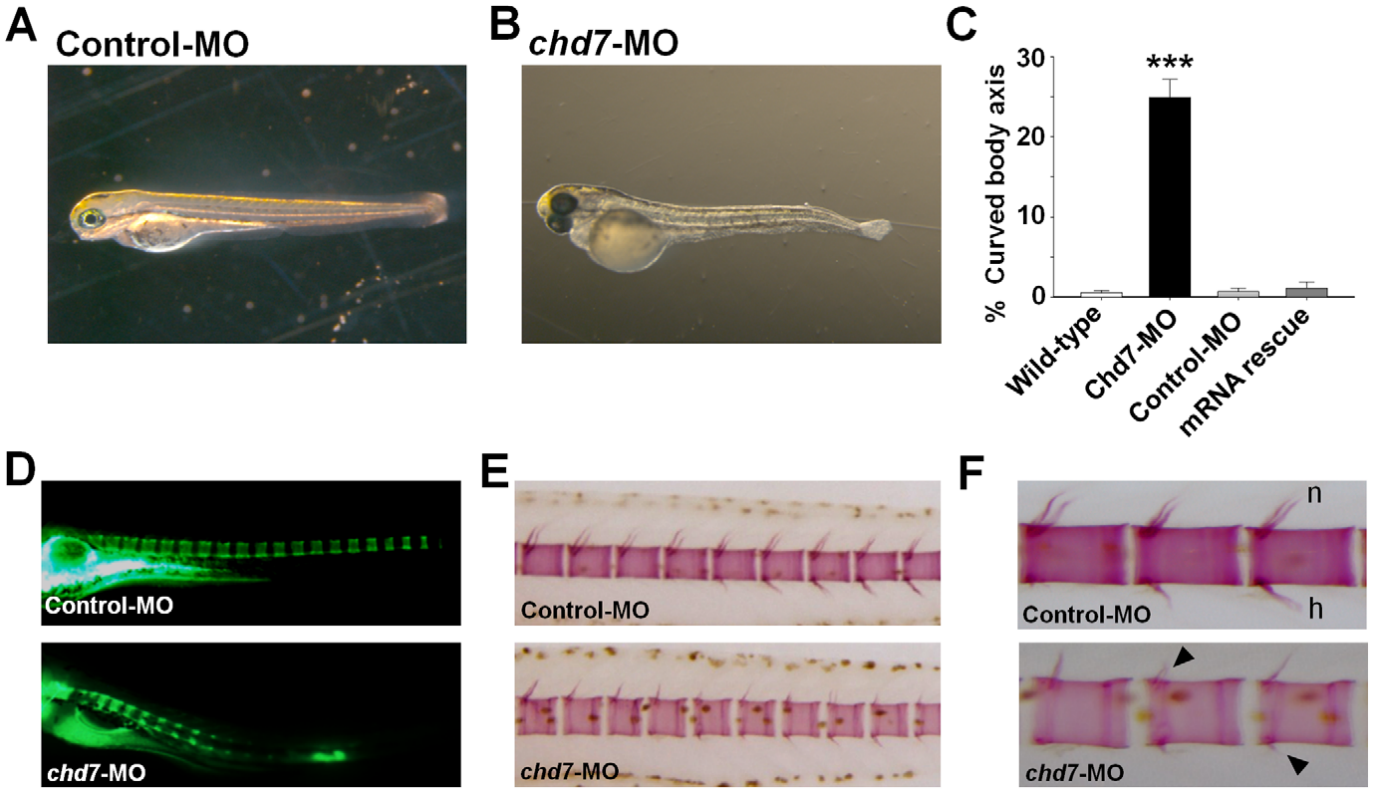
Chd7 deficiency affects vertebral mineralization. Lateral view of control-MO (**A**) and *chd7*-MO-injected (**B**) fish at 3 dpf. *Chd7* morphants exhibited a slight curvature of the long body axis (**B**). A significant percentage of the *chd7*-MO-injected (2 ng/nl) fish had deformed body axis shape (**C**). Lateral views of *chd7* morphants (lower panel) and control siblings (upper panel) at 8 dpf (**D**) and 14 dpf (**E**) showed a reduced bone mineralization. Vertebrae with reduced or no hemal or neural spines were also observed in the *chd7*-MO-injected fish (**F**; black arrow). h, hemal spine; n, neural spine. ***<0.001.

Loss of function of Chd7 led to a pronounced curvature of the body axis in 25% of the morphants (n = 12; p<0.001, ANOVA; Fig. 5 A–C) compared to control-MO (n = 12; Fig. 5 A,C) and wild-type embryos (n = 12; Fig. 5 C). This phenotype was completed rescued by co-injection of *chd7* mRNA (n = 6; Fig. 5 C). Recently, *CHD7* gene polymorphisms were associated with susceptibility to idiopathic scoliosis in human populations [20]. We therefore sought to examine vertebral phenotypes at various developmental stages (Fig. 5 D–F). Chemical staining of the vertebrae at later stages revealed that the spine of zebrafish injected with 2 ng/nl of *chd7*-MO had several skeletal anomalies but exhibited no scoliotic curves (n = 6, 242 fish; Fig. 5 E,F). Notably, *chd7*-MO-injected (2 ng/nl) fish had smaller, irregular vertebral segments (n = 6; Fig. 5 F), wider intervertebral disc space (n = 6; Fig. 5 F) and smaller neural and hemal spines (Arrows; n = 6; Fig. 5 F). The pedicles of the neural spines were in some cases missing or fused. Mineralization can be assessed by the intensity and localization of the alizarin red S stain [21] .We observed a marked decrease in the alizarin red S staining of the vertebral segments of chd7-MO-injected fish (n = 6, 242 fish; Fig. 5 D–F) compared to control-MO ,suggesting a reduction in vertebral mineralization in chd7 morphants . These data suggest *chd7* could play a critical role in vertebral development.

### *Chd7* is required for proper somite polarity and segmental vascularization

**Figure 6:**
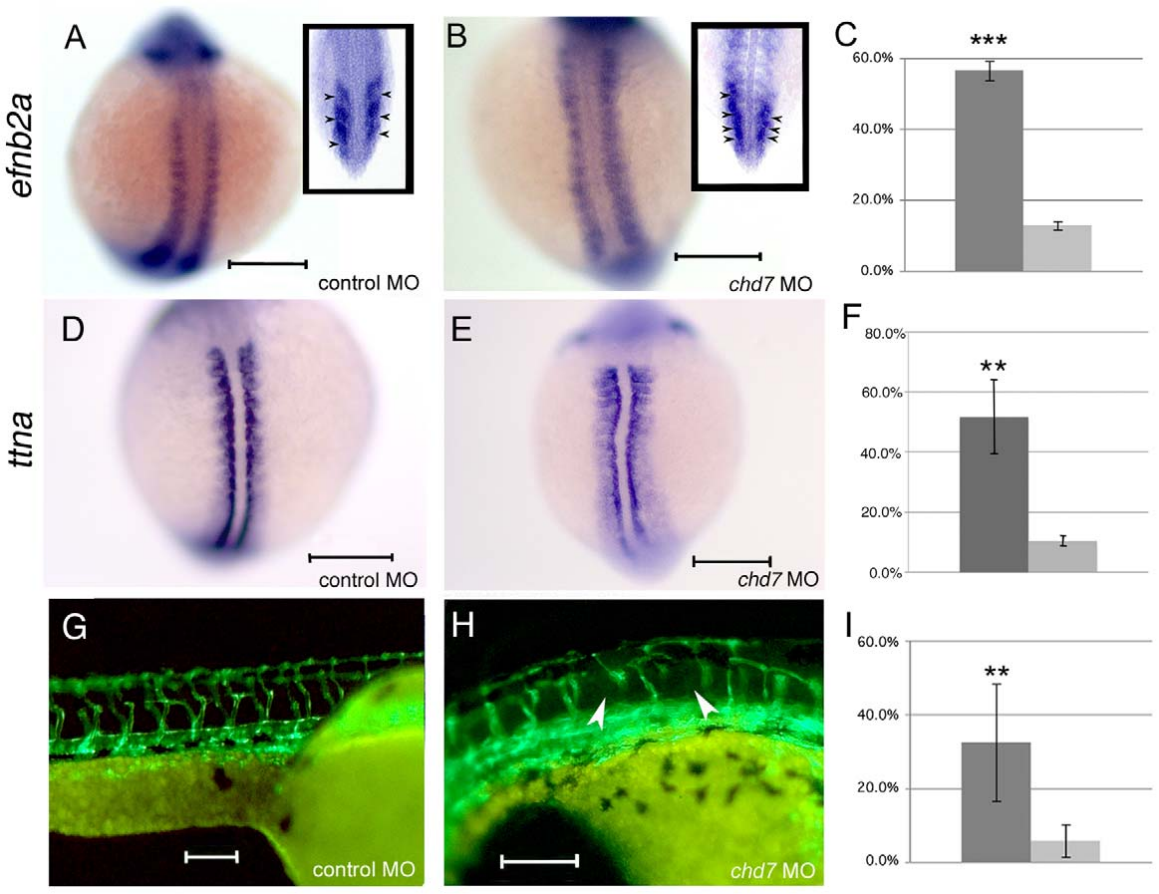
Chd7 knockdown leads to somite boundary and segmental vasculature defects. *Efnb2a* expression in control morphant (**A**) and *chd7* morphant (**B**) zebrafish embryos at the 13-somite stage of development. Top is anterior. Insets in (**A**) and (**B**) represent coronal sections through control or *chd7* morphant embryos, respectively, at the 13 somite stage. Posterior views with dorsal towards the top. Expression of *ttna*, a segment boundary marker reiterates the defects in segment boundary formation in *chd7* morphants (**E**) compared to controls (**D**). Dorsal views with anterior to the top. Segmental vasculature patterning is defective (arrowheads) in *chd7* morphants (**H**) compared to control morphants (**G**) as visualized using *fli1*: GFP transgenic zebrafish at 48 hpf. Sagittal views with anterior to the right. Graphs show percentages of animals exhibiting *efnb2a* expression defects (**C**), *ttna* defects (**F**), or segmental vasculature patterning defects (**I**). The dark gray bar represents *chd7* morphants and the light gray bar represents control morphants. Scale bar: 200 µm. ***<0.001, **<0.01.

Morpholino effects on protein abundance typically last for <7 days [22] and surprisingly, we observed post-embryonic vertebral defects in chd7-MO zebrafish. However, the skeletal malformation observed in chd7-MO injected fish (in Fig. 5) resembles the phenotype observed when somite segmentation is disrupted [23], [24]. To determine whether the abnormality in segmentation of the vertebral axis was due to developmental somite malformation, we analyzed the somite segmentation phenotype. To do so, we examined expression of the segment-polarity gene, *ephrin B2a* (*efnb2a*). *In situ* hybridization expression data for *efnb2a* revealed defects in the delineation of somite borders. To verify this defect in somite borders, a segment boundary marker, *titin a* (*ttna*), was used. Specifically, zebrafish *chd7* morphant embryos showed a loss of distinct borders between individual somites at the 13 somite stage, as exhibited by *efnb2a* expression (146 fish;p = 0.008, t-test; Fig. 6 A–C) and *ttna* expression (112 fish; p = 0.006, t-test; Fig. 6 D–F). Stage matched control MO injected embryos exhibited distinct expression of *efnb2a* within each somite (149 fish, Fig. 6 A–B), and *ttna* expression marked clear boundaries between adjacent somites (143 fish, Fig. 6 D–E). Sectioning of *efnb2a* stained embryos revealed that this defect occurs throughout the somitic tissue in *chd7* morphants (inset, Fig. 6 A–B). Therefore, *chd7* appears to be important for proper segment boundary formation. These results also suggest that the skeletal malformation observed later on during development in *chd7* morphants was likely caused by defective somite segmentation.

Furthermore, since *efnb2a* is involved in arterial-venous differentiation [25] and the development of intersegmental vasculature is dependent on proper segmentation of the presomitic mesoderm (PSM), we utilized a *fli1*: GFP transgenic zebrafish line to test for defects in vascular development caused by Chd7 knockdown. With this resource, we observed aberrant vascular organization. After 48 hours, *chd7* MO injected embryos displayed improper patterning of the intersegmental vasculature along the long axis of the body (56 fish; p = 0.001,t-test; Fig. 6 G–I). Since these vessels form at the boundaries between somites [26], this phenotype is likely due to a lack of intersomite specification in *chd7* morphants, and is similar to defects observed in other mutants defined by aberrant PSM segmentation [25].

### Chd7 function is required for retinal organization

**Figure 7:**
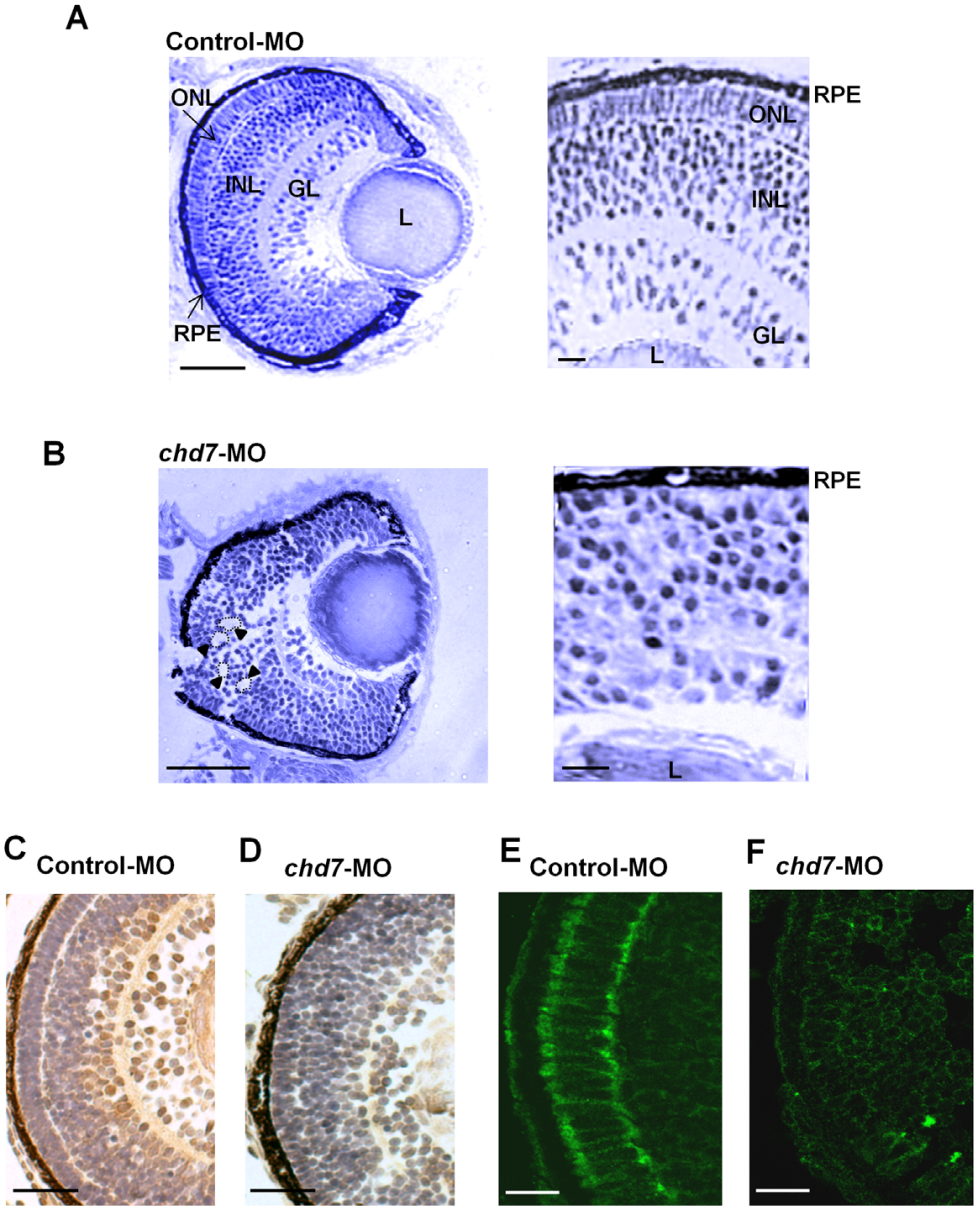
Chd7 plays an essential role in retinal development. Retinal organization of control-MO embryos (**A**) and *chd7* morphants (**B**) was revealed by toludiene blue staining. Compared with the highly organized cells and laminated retinal structure in control-MO fish, *chd7*-MO retinal cells are disorganized (**A–B**: *Left panels*). Retinal lamination defect, including rosette formation,is clearly visible in the *chd7* morphants (examples of rosettes are indicated by dotted lines and arrow heads). GL, ganglion cell layer; INL, inner nuclear layer; L, lens; ONL, outer nuclear layer; R, retina; RPE, retinal pigment epithelium. Scale bar: 50 µm. Zn-8 immunoreactivity was performed to label (brown) retinal gangion cells in control-MO (**C**) and *chd7*-MO embryos (**D**). Scale bar: 30 µm. The expression of retinal ganglion cell-specific marker zn-8 is greatly reduced in *chd7* morphants. The photoreceptor layer of control-MO (**E**) and *chd7*-MO-injected (**F**) embryos were stained with 3A10. Chd7 morphants lacked the photoreceptor layer. Scale bar: 5 µm.

We next thought to further analyze the eye morphology of the *chd7* morphants. To permit a detailed assessment of the *chd7*-MO small eye phenotype, histological analysis was performed on embryos at 72 hpf (Fig. 7). Histology revealed that retinal organization was severely disrupted in *chd7*-MO–injected embryos (120 fish, Fig. 7 B). Specifically, the retinas lacked the characteristic laminated structure of both control-MO (142 fish, Fig. 7 A) and wild-type embryos at 72 hpf. Immunostaining further confirmed disruption of retina formation. Retinal ganglion cells appear to be reduced and disorganized in the *chd7*-MO–injected embryos based on staining for the retinal ganglion marker Zn-8 (n = 5; Fig. 7 C, D). Staining with 3A10, a neurofilament marker was abnormal in that the normally recognizable stripe of expression of the photoreceptor layer was not present in the *chd7*-MO–injected embryos (n = 8; Fig. 7 E, F). Similarly, cone photoreceptor staining with Zpr-1 antibody was absent in *chd7* morphants (Figure S2). These findings suggest a novel role of Chd7 in retinal organization and the development of photoreceptors.

### Chd7 is essential for correct positioning of cranial motor neurons and proper projection of facial and trigeminal motor axons

**Figure 8:**
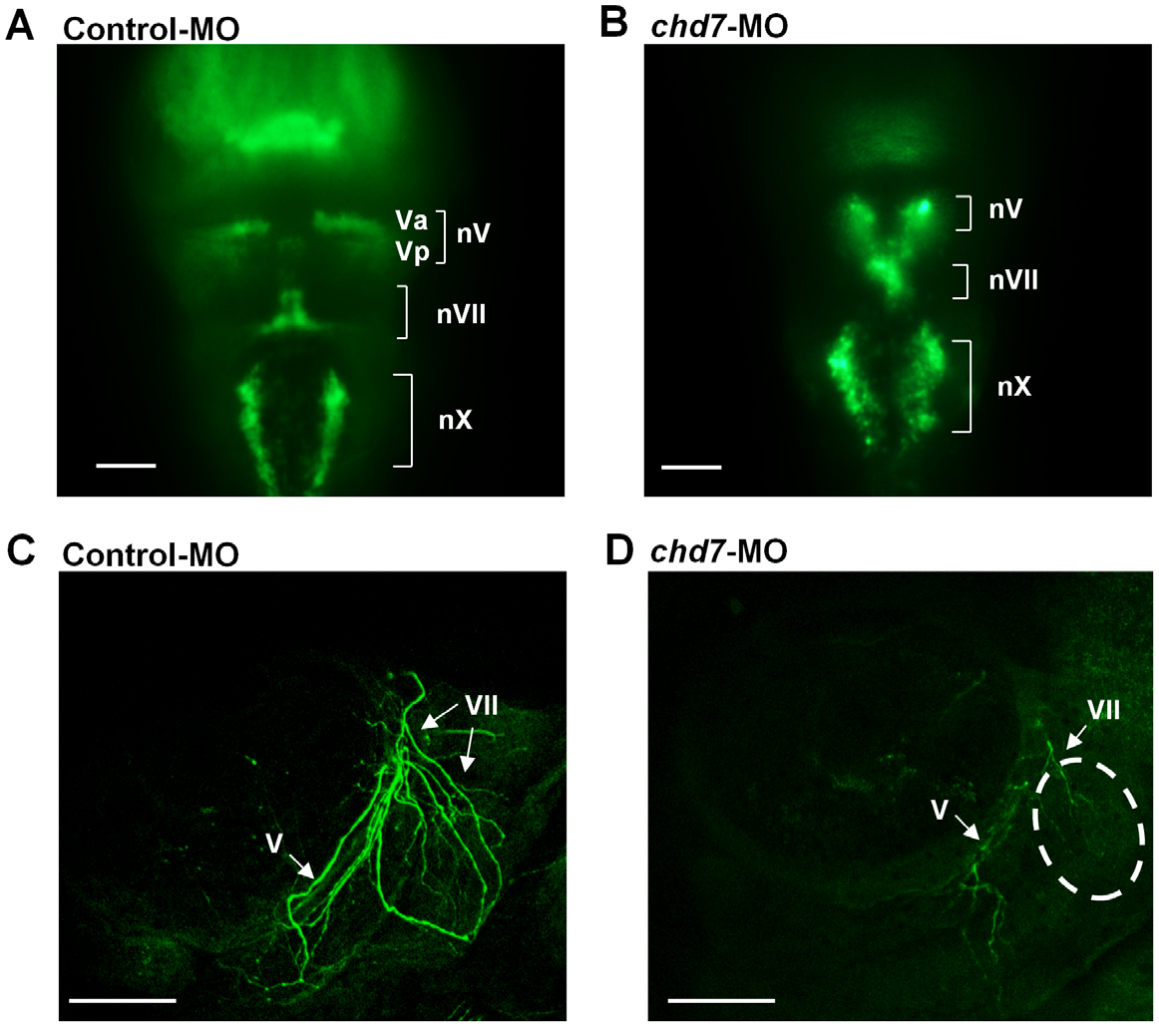
Chd7 is important for cranial ganglia development and proper projection of facial nerves. Dorsal view (rostal towards the top) of confocal fluorescent composite images of hindbrain branchiomotor neurons in control-MO injected (**A**) and *chd7*-MO-injected (**B**) 48 hpf Isl1-GFP transgenic embryos. (**C, D**) Confocal fluorescent composite images showing anti-3A10 antibody-stained axons of 48 hpf embryos. The broken lines indicate the area of significant loss of VII nerves in *chd7* morphants. nV, trigeminal motoneurons; Va, anterior trigeminal motoneurons; Vp, posterior trigeminal motoneurons; nVII, facial motoneurons; nX, Vagal motoneurons; V, trigeminal nerve; VII, facial nerve. Scale bar: 50 µm.

Previous studies have demonstrated a role for Chd7 in neuronal development [27], [28], [29] and patients with CHARGE syndrome often display several neurological disorders. We found that *chd7* is expressed in the developing zebrafish hindbrain and loss of function of chd7 leads to flattening of the head. To determine if these molecules are required for proper neural development in this area, we examined the cranial motor neurons of *chd7*-MO-injected embryos using *Isl1*-GFP transgenic zebrafish embryos. These embryos express GFP under the control of a motor neuron-specific *Isl1* promoter. Interestingly, we observed a disrupted organization of the cranial motor neurons in the *chd7*-MO-injected embryos (n = 9; Fig. 8 B) when compared to control-MO-injected embryos (n = 9; Fig. 8 A). *Chd7*-MO-injected embryos demonstrated a severe disorganization and clustering of both Va and Vp clusters of the trigeminal (nV) neurons (Fig. 8 B). In addition, loss of Chd7 resulted in a reduction of facial (nVII) branchiomotor neuron populations (n = 9; Fig. 8 B). Furthermore, the vagal (nX) motor neurons in *chd7*-MO-injected embryos exhibited aberrant medio-lateral positioning and cell-to-cell spacing (n = 9; Fig. 8 B). *Chd7*-MO-injected embryos also exhibited extremely low levels of staining of the peripherally extending axons from nV neurons when compared to control-MO-injected embryos (n = 9; Fig. 8 C, D). Furthermore, *chd7*-MO injected embryos exhibited severe loss of the facial sensory ganglion and the axonal extensions from nVII neurons (n = 9; Fig. 8 C, D).

### Chd7 plays a key role in cranial neural crest development

**Figure 9:**
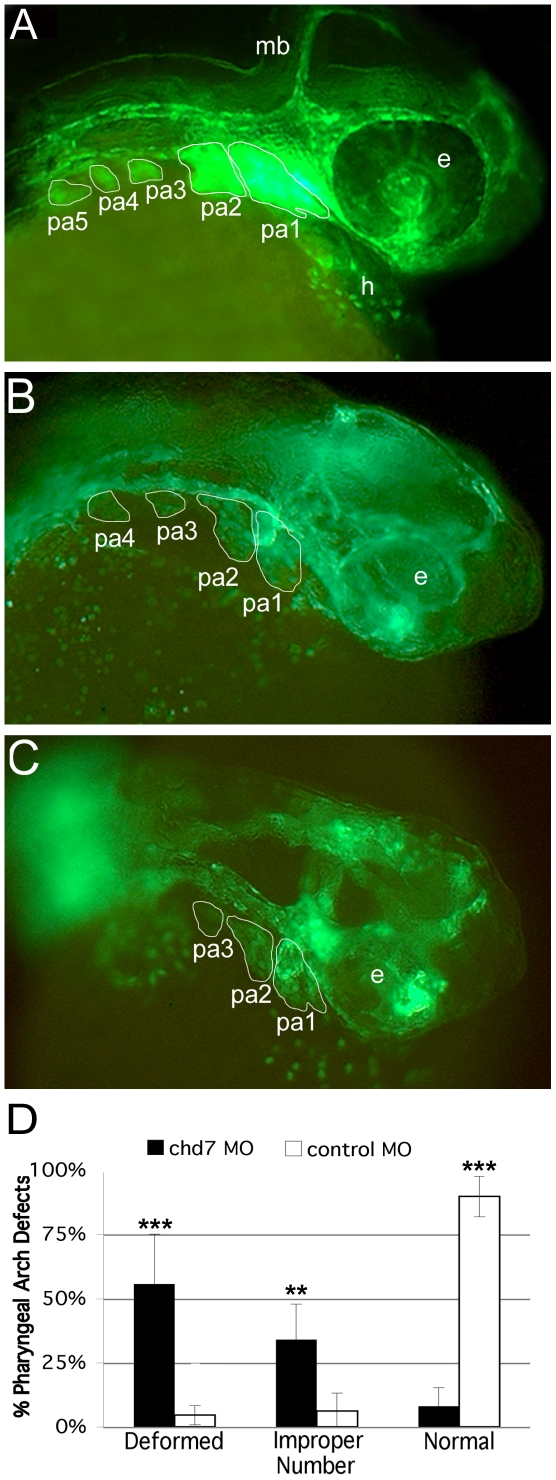
Knockdown of *chd7* results in defects in CNC development. Fli1-GFP transgenic embryos were used for MO injections to determine whether Chd7 depletion had an effect on CNC development. Embryos were injected at the 1 cell stage and examined for effects at 34–36 hpf. (**A**) Control-MO injected zebrafish embryos did not exhibit defects in CNC segment shape or number. Alternatively, the majority of *chd7*-MO-injected embryos exhibited aberrant CNC development including an improper number of CNC segments (**B**), as well as severely disorganized (**C**) or missing CNC populations. (**D**) Based on the t-test comparing means, there was a significant difference in CNC defects between *chd7*-MO and control-MO-injected zebrafish embryos. More than half of the *chd7*-MO-injected fish had deformed or missing CNC segments and about one third developed an improper number of segments. ** = 0.01; ***<0.001; e, eye; mb, midbrain; pa1–5, pharyngeal arches 1–5.

CHARGE syndrome is thought to result from the abnormal development of the neural crest. Recently, CHD7 and PBAF have been shown to cooperate during embryonic development to promote proper neural crest specification and cell migration [17]. We sought to examine the relationship between Chd7 and CNC development in zebrafish using a Fli1-GFP transgenic zebrafish line. We injected Fli1-GFP embryos with *chd7*-MO at the single cell stage and then assayed for CNC defects between 34–36 hpf by counting the number of CNC segments, which should approximate pharyngeal arch number, within each embryo. We observed CNC defects in over 90% of *chd7*-MO-injected embryos (63 fish, Fig. 9 D). About one third of these had a reduction in the number of CNC segments, whereas the remaining animals showed more severe phenotypes characterized by the gross disorganization or absence of obvious CNC populations (Fig. 9 B, C). Disorganized CNC segments tended to possess fewer cells and lacked sharp borders between adjacent segments (Fig. 9 C). The phenotype of control-MO-injected zebrafish embryos mimicked that of wild type animals including well-defined segments and appropriate segment numbers (71 fish, Fig. 9 A). These data lend support for the function of Chd7 in proper CNC development and patterning. Whether the defects reported here are due to aberrant CNC migration or improper pharyngeal arch patterning remains to be investigated.

## Discussion

The present results show a novel and essential role of Chd7 in retinal organization, axial patterning and bone development and mineralization. Furthermore, knockdown of zebrafish *chd7* results in abnormalities similar to those described in CHARGE Syndrome, as we observed inner ear, heart, eye, cranial ganglia, neural crest and skeletal defects in *chd7* morphants. Collectively, our findings demonstrate that Chd7 function is essential for proper neural and cranial neural crest development. In addition, to our knowledge, this is the first study to report the requirement of *chd7* in retinal and vertebral development.

Chd7 has been implicated in spinal deformities [20] and scoliosis is one of the minor clinical features in some CHARGE patients [11]. Here, loss of function of Chd7 resulted in the curvature of the body axis, irregular vertebral segments and smaller neural and hemal spines. The *chd7* morphants did not exhibit any skeletal defects reminiscent of a scoliotic adult phenotype, however, suggesting that either scoliosis in CHARGE patients is secondary to the effects of attenuated Chd7 levels, or due to primary effects that occur later in development. Our findings also showed that zebrafish embryos deficient in Chd7 mineralize vertebrae more slowly than control siblings, indicating a critical role for Chd7 in promoting mineralization during normal development. Delays in vertebral mineralization may result from a general retardation in growth and development; however, we did not detect any distinguishable differences in length between the *chd7* morphants and controls at all developmental stages examined (Table S1). Interestingly, a similar delay of mineralization in zebrafish has been reported after knockdown of *collagen XXVII* [30]. Furthermore, in the absence of BMP signaling, zebrafish bone mineralization was delayed [31]. Chd7 has been shown to regulate BMP signaling [27], [32]. Thus, it is possible that disruption of Chd7 affected the transcription of genes that are crucial for bone mineralization. We also show that a loss of somite border identity during somitogenesis, as exhibited by *efnb2a* and *ttna* expression, is linked to a loss of organized segmental vasculature following *chd7* MO injection and skeletal defects. We hypothesize that early defects in *efnb2a* expression lead to improper segmental patterning later in development, affecting structures such as segmental vasculature and bone mineralization. The link between the process of somitogenesis and the defects in border specification will require further investigation. For example, since *efnb2a* expression persists after somite epithelialization, the irregular *efnb2a* expression observed here could be the result of an accumulation of upstream defects occurring early in the somitogenesis signaling pathway (i.e., indirect effects). Alternatively, it is also possible that Chd7 modulates *efnb2a* expression in a manner that is independent of the process of somite formation (i.e., direct effects).

CHARGE syndrome patients often have microphthalmia [33], [34], [35] and here we found that loss of function of Chd7 in zebrafish resulted in a small eye phenotype. The retinas in zebrafish develop rapidly and begin differentiation by 36 hpf [36], [37]. The differentiating retinal neurons undergo terminal differentiation by 52 hpf and become functional at around 3 dpf [37], [38], [39]. A functional retina consists of a laminated neuronal tissue with the ganglion cell layer on the inside, inner nuclear layer in the middle, and the outer nuclear layer on the outside. In between these layers are the synaptic layers: the inner and outer plexiform layer [36], [40]. Notably, the retinas in *chd7* morphants fail to laminate, which suggests a novel function for Chd7 in retinal patterning. Furthermore, our findings are similar to disruptions in retinal lamination as observed in the young zebrafish mutant that carries a mutation in *brg1*, a gene that encodes for a subunit of the Swi/Snf of ATP-dependent chromatin remodeling complexes [37]. Moreover, retinal ganglion cells were reduced and the photoreceptor layer were absent in the *chd7*-MO-injected embryos. These data suggest that Chd7 is required for the development of retinal cells. In *Drosophila*, the *chd7* ortholog *kismet* is essential for transcription of the pro-neural factor *atonal* (*Atoh1*) and regulation of retinal photoreceptor cell development [41]. *Drosophila ato* mutants produce no photoreceptors in the eye [42]. Chd7 has also been recently shown to control the expression of *sox9* [17]. Zebrafish *sox9* mutants reveal that Sox9 is required for retinal differentiation and it also helps with retinal organization and regulates the number of photoreceptor cells [43]. Thus, it is likely that that loss of function of Chd7 disrupts the gene network that is crucial for retinal cellular development and organization.

Several ganglionic defects have been reported in CHARGE patients, including defects in the facial and vestibulo-acoustic ganglia [44], [45]. *Chd7* knockdown resulted in important phenotypes relevant to cranial ganglia development, branchiomotor development, and vagal motor neuron positioning. Anomaly in facial nerves has also been demonstrated in CHARGE syndrome [5]. Loss of function of Chd7 resulted in a severe loss of facial nerves in the morphants. Altogether, these results suggest that Chd7 and downstream targets are important for neuronal development and proper axonal projections.

Neural crest cells are multipotent cell populations with the ability to migrate, leading to the formation of several key developmental structures including bones, cartilages, nerves, and connective tissues [46]. CNC, specifically, migrate into the pharyngeal arches where they play a role in the formation of facial bone, muscle, and cartilage [47]. In addition, CNC migrate into the optic vesicle and otic placode, where they play key roles in the development of muscular and skeletal elements in the eye and the inner ear, respectively [48], [49]. Some key developmental defects in CHARGE patients can be traced to aberrant CNC development, including coloboma of the eye, external ear malformations, inner ear defects and a spectrum of facial defects [6], [50], [51]. Our *chd7* morphant zebrafish embryos displayed obvious defects in CNC migration, based on observations of pharyngeal arch development and number. We did not fully characterize CNC activity in the optic nerve or otic placode of our *chd7* morphants, however, we did observe and quantify significant defects in eye and otic vesicle development. CHD7 is essential for the formation of multipotent migratory neural crest in humans and *Xenopus* [17] and recently, CHD7 was found to play a role in proper neurogenesis of the inner ear in mice [27]. Our results support a role for Chd7 in proper CNC migration and differentiation, and substantiate the use of *chd7* morphant zebrafish as an *in vivo* animal model for CHARGE Syndrome.

The *chd7* –MO-injected embryos displayed a very specific defect in otolith and semicircular canal formation and they also exhibited a circling swimming behavior consistent with vestibular dysfunction. Such inner ear malformations are very similar to those reported in CHARGE syndrome patients [52], [53], [54], [55]. In addition, CHARGE patients have been reported to have vestibular problems and hyperactivity [56], and this may be due to inner ear defects. CHD7 is necessary for proliferation of inner ear neuroblasts and inner ear morphogenesis by the maintenance of *Fgf10*, *Otx2* and *Ngn1* expression [27]. Fgf signaling and *Otx* genes have also been shown to be important for inner ear development in zebrafish [57], [58], [59], [60]. Thus, our zebrafish model for CHARGE syndrome can be useful to further explore the mechanisms underlying inner ear anomalies in CHARGE pathogenesis. For instance, it will be interesting to investigate whether loss of function of *chd7* in zebrafish results in a downregulation of *Fgf10* and *Otx2* and whether overexpression of one or more of these genes could rescue *chd7* –MO phenotypes.

Heart defects have been reported to be associated with CHARGE syndrome [2], [3], [5], [61], [62]. Here we report that the hearts of *chd7*-MO-injected embryos are developmentally impaired and have signs of severe pericardial edema. Heart anomalies described in CHARGE syndrome patients include ventricular and atrial septal defects and conotruncal defects [61], [63]. In this study, we did not fully characterize the observed heart defects in the *chd7* morphants. The atrial and ventricular chambers in these fish can be distinguished, but these compartments were dysmorphic and more tube-like than the well-defined and tightly looped chambers of wild-type fish. Future studies using *chd7* zebrafish morphants could help to identify components in the Chd7 regulatory network that are essential for heart development and further our understanding of one of the major anomalies reported in CHARGE patients.

In conclusion, we have examined the role of zebrafish Chd7. We provide evidence that zebrafish highly express *chd7* in the retina, brain and somite boundaries. Loss of function of Chd7 resulted in several morphological defects similar to those observed in patients with CHARGE syndrome. We then performed a detailed analysis of uncharacterized defects of CHARGE syndrome and show that the presence of Chd7 is crucial for proper neural, retinal and vertebral development in zebrafish. These data provide new insights on the role of Chd7 and the mechanistic link between defects in the Chd7 gene and the organs and systems dysfunction associated with CHARGE syndrome. Furthermore, on the basis of the overlap in clinical features between zebrafish *chd7* morphants and CHARGE syndrome, we suggest zebrafish can be a valuable *in vivo* tool to further understand the pathophysiological mechanisms underlying the abnormalities associated with CHARGE syndrome.

## Materials and Methods

### Animals

Wild-type AB, *Isl1*-GFP WIK transgenic and *Fli1*-GFP transgenic zebrafish (*Danio rerio*) embryos were raised at 28.5°C, and collected and staged using standard methods [64]. Wild-type AB stains fish and *Fli1*-GFP transgenic zebrafish were purchased from the Zebrafish International Resource Center (ZIRC; University of Oregon, Eugene, OR). *Isl1*-GFP WIK transgenic line was kindly provided by Dr. Hitoshi Okamoto (RIKEN Brain Science Institute, Wako, Japan) [65]. Embryos and larvae were anaesthetized in 0.02% tricaine (MS-222; Sigma Chemical, St. Louis, MO) in phosphate-buffered saline (PBS) prior to all procedures.

### Ethics Statement

All protocols were carried out in compliance with the guidelines stipulated by the Canadian Council for Animal Care (CCAC), the CHU Sainte-Justine Research Center, as well as the Institutional Animal Care and Use Committee (IACUC) at Syracuse University and at the University of Montreal. This study was approved by the CHU Sainte-Justine Research Center ,University of Montreal (ZF-09-60/Category B) and the Syracuse University (IACUC # 07-010) ethics committees.

### Design and synthesis of DIG-labeled RNA probes for *chd7*, *efnb2a* and *ttna*

Total RNA was isolated from blastula stage zebrafish embryos using the RNeasy Mini Kit (Quiagen, Valencia, CA). Reverse transcription was performed to make cDNA using the QuantiTect Reverse Transcription Kit (Quiagen, Valencia, CA). *Chd7* fragments were amplified by PCR using 5′-GCTATTGACCGCTTCTCTCG-3′ and 5′-TGCTCCTTTACGCAGGAGAT-3′ primers (resulting in a 362 bp amplicon from about halfway through the cDNA sequence) and cloned into the pGEM-T Easy vector (Promega, Madison, WI) following the manufacturer's protocol. Zebrafish *efnb2a* [66] and *ttna* [67] plasmids were kind gifts from Dr. Steven Wilson and Dr. Andrew Oates respectively. To synthesize the antisense probe, the previously constructed plasmid was linearized using the restriction enzyme SpeI (New England BioLabs, Inc., Ipswich, MA) for 2 hours at 37°C. Linearization was confirmed by gel electrophoresis using a 1% agarose gel. Linearized plasmid was incubated with T7 RNA Polymerase (Roche Diagnostics, Indianapolis, IN) in the presence of digoxigenin (DIG) label (Roche Diagnostics, Indianapolis, IN) and RNase Inhibitor (Roche Diagnostics, Indianapolis, IN) for 2 hours at 37°C. To synthesize the sense strand, the restriction enzyme ApaI (New England Biolabs, Inc., Ipswich, MA) and SP6 RNA Polymerase (Roche Diagnostics, Indianapolis, IN) were used. Plasmid DNA was eliminated with DNase (Roche Diagnostics, Indianapolis, IN) by incubation at 37°C for 20 minutes. DIG-labeled RNA was precipitated in 0.2 M EDTA, 4 M LiCl, and 100% ethanol overnight at −20°C, and then resuspended in DEPC water. Agarose gel electrophoresis was used to confirm the presence of a purified probe and any unused probe was stored at −20°C.

### Whole mount *in situ* hybridization analysis

Whole mount *in situ* hybridization was performed on staged zebrafish embryos using both sense and antisense *chd7*, *efnb2a* and *ttna* riboprobes. Methods for *in situ* hydridization analysis followed [68], [69]. Briefly, staged embryos were fixed overnight in 4% paraformaldehyde, and then dehydrated in methanol. When ready to use, embryos were rehydrated in phosphate buffered saline with 0.1% Tween-20 (PBSt). Embryos were permeabilized by proteinase K digestion and then hybridized with the riboprobes overnight at 70°C. The next day, embryos were put through graded solutions of 75%, 50%, and 25% prehybridized solution in 2× saline-sodium citrate (SSC) followed by a wash in 0.2× SSC for 30 minutes at 68°C. They were then placed in blocking solution for several hours, and incubated in α-DIG antibody overnight. Finally, embryos were washed again and incubated in staining solution in the dark until sufficient staining appeared on the embryos. Embryos were dehydrated in methanol to facilitate clearing of background staining and then rehydrated in PBSt. Embryos were stored in glycerol.

Embryos in glycerol were visualized using a Zeiss M2 Bio Stereomicroscope, with motorized focus drive and X-Cite UV light source with GFP filter. Images were captured using a Zeiss Axiocam digital camera connected to an Antec PC and processed with Adobe Photoshop 7.0.

### Generation of zebrafish *chd7* morpholino and mRNA rescue experiments

To eliminate Chd7 function, two types of antisense morpholino oligonucleotide were used to disrupt the translation of *chd7* transcripts. The translation-blocking morpholino, 5′-TGCAGCCAAGCTTAGAAGCAGGAC-3′ and the splice blocking, 5′-TTATTTTCTGGCACTAACCATGTCC-3′ were synthesized by Gene Tools (Philomath, OR). The morpholino was injected into single-cell stage zebrafish embryos at doses of 2 ng/embryo, 4 ng/embryo and 6 ng/embryo. To validate the function of the *chd7* splice blocking morpholino, RT-PCR was used to check for improper splicing using the following primer set: forward- 5′-AGGTGGACTCCGAAGGAAAC-3′, reverse- 5′-CCGTCATCACCACATTTGAG-3′. Amplified cDNA was visualized using gel electrophoresis.

To confirm the specificity of the chd7 morpholinos, a mismatch morpholino (lower case) was used: TGgAcCCAAcCTTAcAAcCAGGAC (Gene Tools. OR). Furthermore, rescue experiments were performed. Full-length wild type zebrafish chd7 gene was subcloned into pcs2+ vector and capped mRNA was synthesized using the Sp6 promoter and the mMessage mMachine Kit (Ambion). Three hundred picograms of the synthetic mRNA was then injected into embryos at the one-cell stage. Rescued embryos were visualized and counted using the Olympus SZX12 stereoscope.

Injected and uninjected embryos were then incubated in embryo media at 28.5°C for 24 h, after which they were assessed for viability. Wild type, Isl1-GFP WIK and Fli1-GFP transgenic zebrafish embryos injected with *chd7* morpholino were assessed for morphological differences from control morpholino injected or uninjected embryos under an Olympus SZX12 stereoscope at 24, 48 and 72 hpf. Isl1-GFP transgenic zebrafish were stage matched to 48 hpf and fixed in 4% paraformaldehyde for 4 h and then analyzed for differences in branchiomotor neuron development and migration from uninjected controls using a Zeiss AxioImager Z1 compound microscope. Images were photographed using a Zeiss LSM 510 confocal microscope at 488 nm under a 20× objective, and were compiled using Zeiss LSM Image Browser software. Fli1-GPF zebrafish were staged to 34–36 hpf and screened for CNC segmentation defects using an X-Cite UV light source with GFP filter mounted to a Zeiss M2 Bio Stereomicroscope.

### Immunohistological procedures

Zebrafish specimens were fixed in 4% paraformaldehyde and were either used for whole-mount immunostaining or embedded in paraffin. Transverse sections (1.5–3 µm) of paraffin-embedded specimens were deparaffinized in xylene and were rehydrated in a graded series of ethanol. Serial sections were collected from the central retina.

For immunofluorescence microscopy, whole embryos and eye sections were washed several times in PBS and permeabilized for 30 min in 4% Triton-X 100 containing 2% bovine serum albumin (BSA) and 10% goat serum. Following permeabilization, tissues and retinal sections were incubated in the primary antibody 3A10 (Developmental Studies Hybridoma Bank; 1∶500) for 48 h at 4°C on a shaker. Tissues and eye sections were washed several times in PBS over a 24 h period, and then incubated in the secondary antibody conjugated with Alexa Fluor 488 (Molecular Probes, Carlsbad, CA, 1∶2000) for 4–6 h at room temperature. Animals were washed in PBS several times, de-yolked, cleared in 70% glycerol and mounted. Z-stack images were photographed using a Zeiss LSM 510 confocal microscope under a 20× objective, and were compiled using Zeiss LSM Image Browser software.

For immunoperoxidase methods, slides were incubated with a blocking serum (Vectastain; Vector Laboratories, Burlingame, CA) for 45 minutes, after which they were blotted and then overlaid with the primary antibodies Chd7 (Santa Cruz Biotechnology, Santa Cruz, CA ; 1/600) and zn-8 (Developmental Studies Hybridoma Bank; 1/200) for 18 hours at 4°C. The slides were washed 3 times in PBS, pH 7.4, and incubated with secondary antibodies (anti- mouse (1∶1000) or anti- rabbit (1∶1000), Vectastain) for 1 hour at room temperature, followed by staining according to the avidin-biotin-peroxidase complex method (Vectastain ABC assay). Color was developed with 3,3-diaminobenzidine (Dako Diagnostics Inc., Mississauga, ON, Canada) containing hydrogen peroxide. Slides were counterstained with Harris modified hematoxylin (Fisher Scientific, Ottawa, ON, Canada). Eye sections were also stained with 1% Toluidine Blue. Images were captured using a Leica DMR microscope mounted with a Qimaging Retiga 1300 camera.

### Skeletal staining

Juvenile zebrafish were fixed in 4% paraformaldehyde in PBS for 48 hr at 4°C and dehydrated to 100% ethanol over 2 days. Fish were then cleared in a 1% KOH solution containing approximately 4.5 ml of 3% H_2_O_2_ per 100 ml 1% KOH for 2 h, followed by 2 h in half this concentration (2.25 ml H_2_O_2_ per 100 ml 1% KOH). This latter step ensures a stepwise progression from H_2_O_2_ to water, which helps reduce tissue expansion. Zebrafish were then rinsed twice in distilled water, and transferred to a 30% saturated sodium tetraborate solution (Borax; Acros Organics) in water, overnight. Any remaining soft tissues were digested in a solution containing 1.0% trypsin and 2.0% Borax in water for 2–4 h. Digestion was deemed complete when the primary axis of the axial skeleton became visible. The fish were subsequently rinsed three times in distilled water to remove all residual trypsin, prior to staining for 12 h or more in a solution of 75% ethanol and Alizarin red S. Vertebral mineralization was assessed after Alizarin red S staining. Fish were examined using a Leica M205FA stereomicroscope. Digital images were collected with Leica DFC490 camera.

Calcein staining was performed as described [70]. Briefly, fish were anesthetized in 0.6 mM MS-222 buffered to pH 7.0 and immersed in 0.2% calcein in 10% Hank's solution buffered to pH 7.2 for 10 min followed by three 10 min washes in Hank's solution. Vertebral mineralization was assessed after calcein staining using a Leica DMR microscope. Digital images were collected with a Qimaging Retiga 1300 camera.

### Sample number and statistical analysis

The sample number (n) refers to the batch number and each batch number consisted of at least 40 fish. For each data set, we provided the batch number followed by the total fish number used. The sample number for wild-type fish consisted of 12 batches with a total of 510 fish used (n = 12,510 fish); 12 batches with a total of 436 fish for *chd7* morphants (n = 12,436 fish); 12 batches with a total of 498 fish for control-MO injected embryos (n = 12, 498 fish) and 6 batches with a total of 249 mRNA rescue experiments (n = 6, 249 fish). Immunohistochemistry were performed on at least 6 batches for both control-MO and *chd7*-MO fish. Statistical analyses were performed and data were plotted in SigmaPlot 11.0 (Systat Software Inc., San Jose, CA, USA). Significance was determined using paired Student's *t*-tests, one-way anovas and Fisher's least significant difference tests for normally distributed, equal variance data. Kruskal–Wallis anova and Dunn's method of comparison were used for non-normal distributions.

## Supporting Information

Figure S1 ***Chd7*** **- MO injections are dosage dependent.** As the concentration of the *chd7*-MO injection increased, the phenotypic defects became more severe. (**A**) Control-MO injected zebrafish showed no phenotypic defects 48 hpf and were comparable to wild type zebrafish at the same age. Embryos injected with 2 ng/nl (**B**), 4 ng/nl (**C**), or 6 ng/nl (**D**) *chd7*-MO showed increasing severity of developmental defects with increasing MO concentration.
(TIF)

Figure S2 **Chd7 plays an essential role in photoreceptor development.** The photoreceptor layer of control-MO (**A**) and *chd7*-MO-injected (**B**) embryos were stained with Zpr-1. Chd7 morphants lacked the photoreceptor layer.
(TIF)

Table S1 **Measurement of zebrafish size at different ages.**
(TIF)
