# Supplementary figures and images for: Role of Chd7 in Zebrafish: A Model for CHARGE Syndrome
Source: PLoS One. 2012 Feb 20;7(2):e31650. doi: 10.1371/journal.pone.0031650 (PMC3282775; doi:10.1371/journal.pone.0031650)

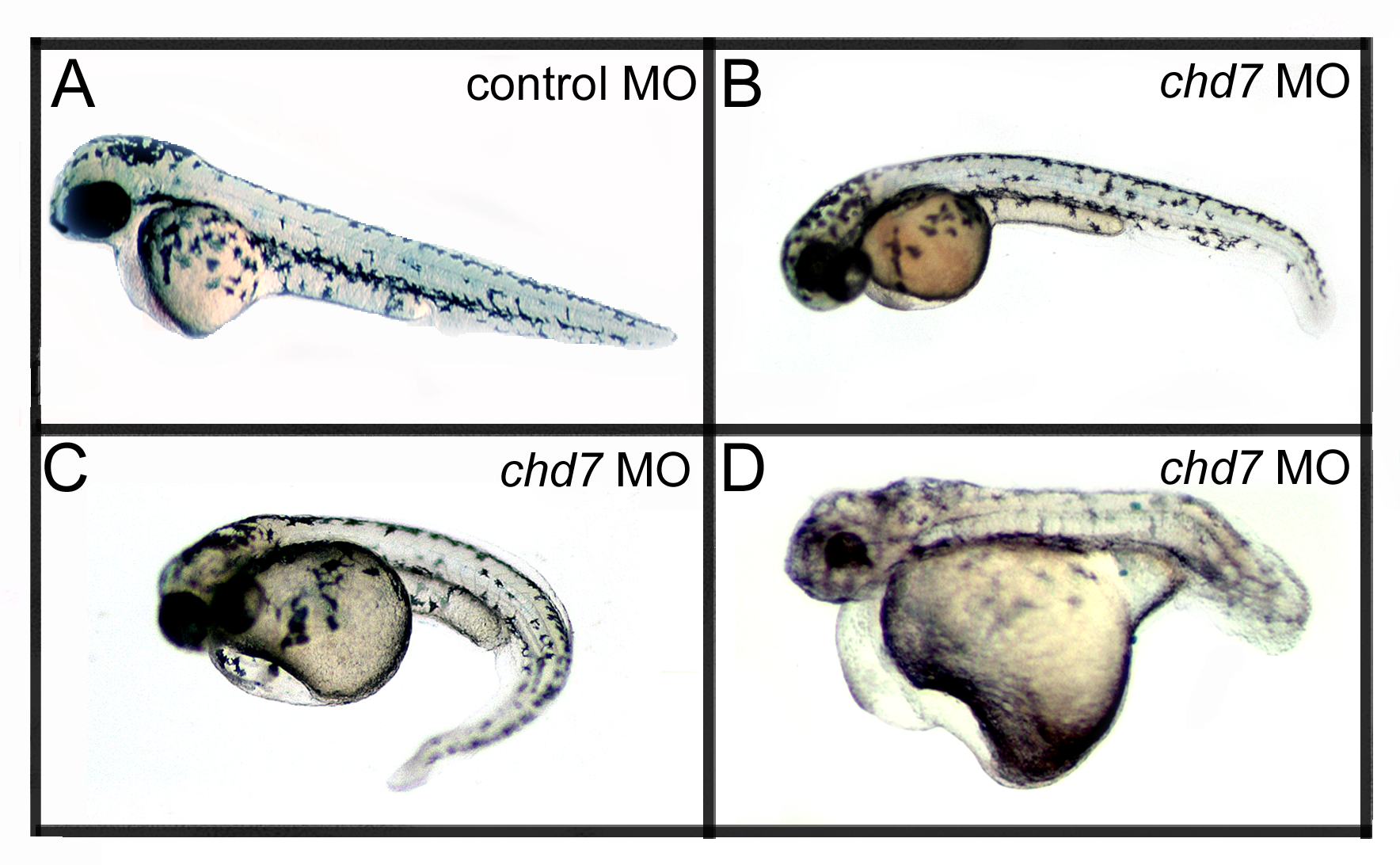

Supplement: Figure S1 — Chd7 - MO injections are dosage dependent. As the concentration of the chd7-MO injection increased, the phenotypic defects became more severe. (A) Control-MO injected zebrafish showed no phenotypic defects 48 hpf and were comparable to wild type zebrafish at the same age. Embryos injected with 2 ng/nl (B), 4 ng/nl (C), or 6 ng/nl (D) chd7-MO showed increasing severity of developmental defects with increasing MO concentration. (TIF) [file pone.0031650.s001.tif]

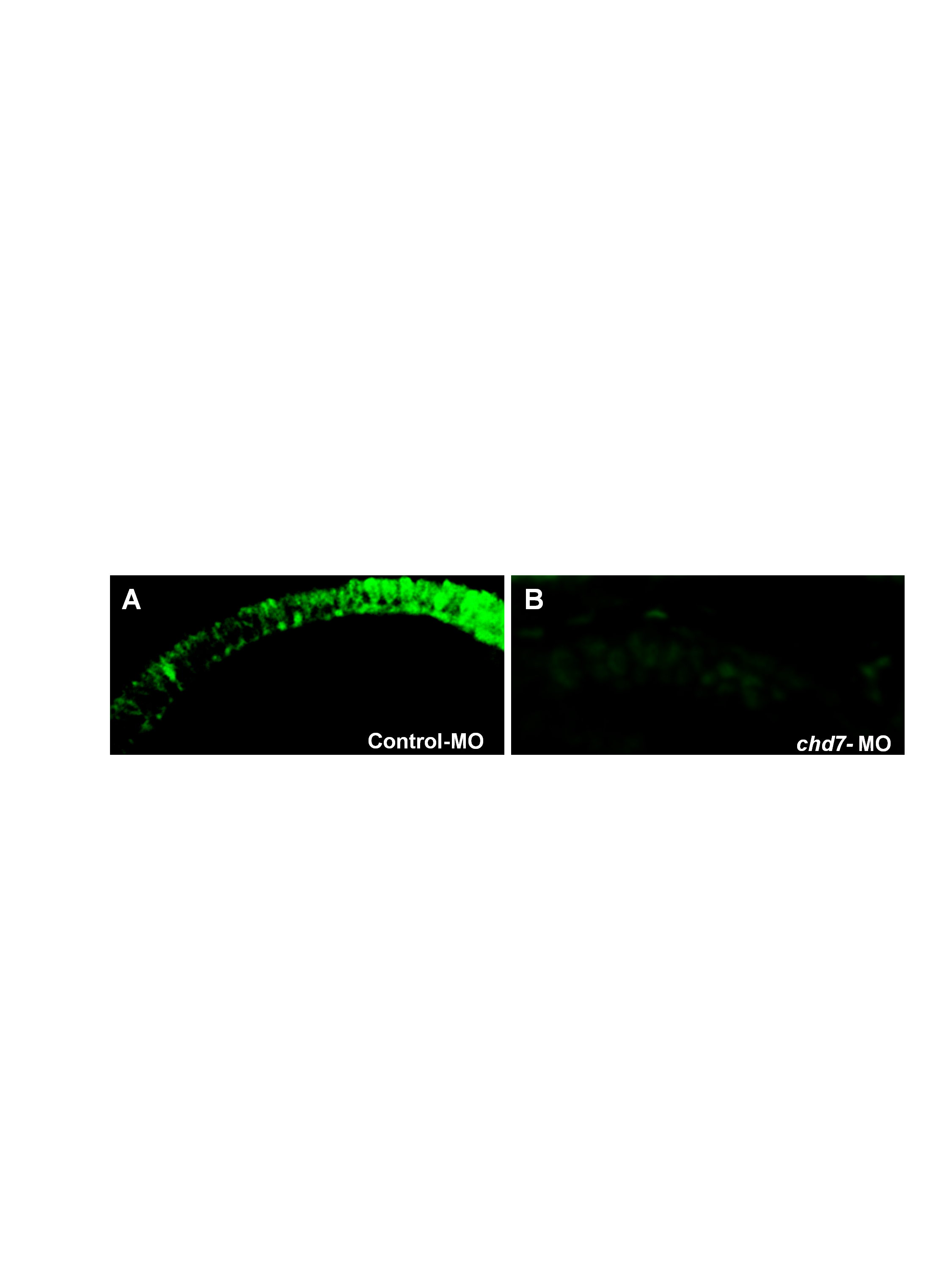

Supplement: Figure S2 — Chd7 plays an essential role in photoreceptor development. The photoreceptor layer of control-MO (A) and chd7-MO-injected (B) embryos were stained with Zpr-1. Chd7 morphants lacked the photoreceptor layer. (TIF) [file pone.0031650.s002.tif]

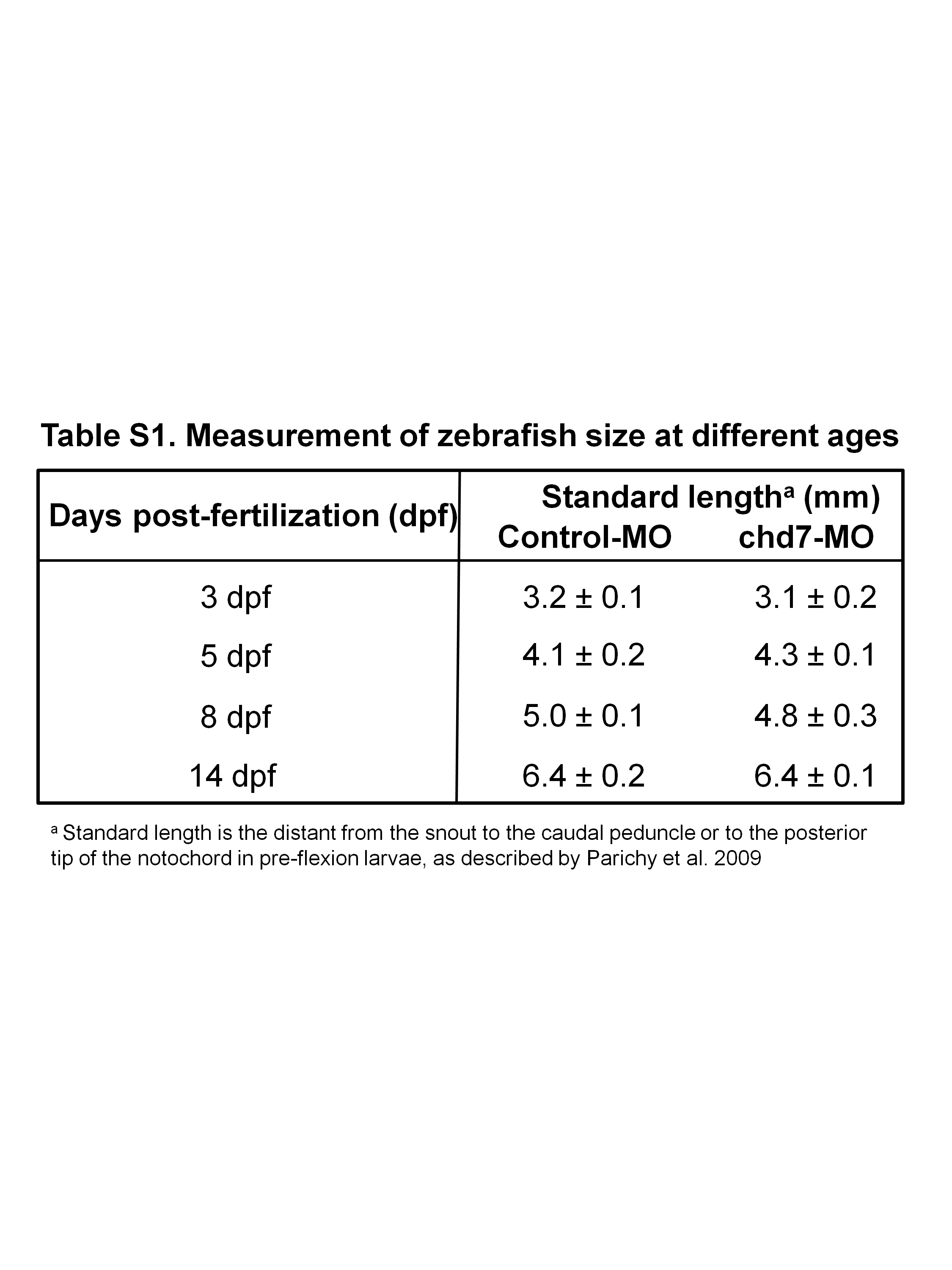

Supplement: Table S1 — Measurement of zebrafish size at different ages. (TIF) [file pone.0031650.s003.tif]
